# Morphological and Functional Analysis of Residual Lung After Pneumonectomy in Lung Cancer Surgery via 3D-CT Method

**DOI:** 10.3390/life15081265

**Published:** 2025-08-10

**Authors:** Omer Topaloglu, Rıza Aktepe, Kubra Nur Kilic, Sami Karapolat, Ali Yavuz Uzun, Elvan Senturk Topaloglu, Atila Turkyilmaz, Serkan Ozden, Aziz Gumus, Celal Tekinbas, Hasan Turut

**Affiliations:** 1 Department of Thoracic Surgery, Faculty of Medicine, Recep Tayyip Erdogan University, 53100 Rize, Turkey; hasan.turut@erdogan.edu.tr; 2 Department of Anatomy, Faculty of Medicine, Recep Tayyip Erdogan University, 53100 Rize, Turkey; riza.aktepe@erdogan.edu.tr; 3 Department of Thoracic Surgery, Faculty of Medicine, Karadeniz Technical University, 61100 Trabzon, Turkey; kubranurseyis@yahoo.com (K.N.K.); samikarapolat@yahoo.com (S.K.); atilaturkyilmaz@hotmail.com (A.T.); serknzdn@gmail.com (S.O.); celaltekinbas3@hotmail.com (C.T.); 4 Department of Anatomy, Faculty of Medicine, Karadeniz Technical University, 61100 Trabzon, Turkey; aliyavuzuzun@ktu.edu.tr; 5 Department of Pulmonology, Faculty of Medicine, Recep Tayyip Erdogan University, 53100 Rize, Turkey; elvan.senturktopaloglu@erdogan.edu.tr (E.S.T.); aziz.gumus@erdogan.edu.tr (A.G.)

**Keywords:** pneumonectomy, 3D computed tomography (3D-CT), lung volume calculation, pulmonary function test, residual lung expansion

## Abstract

**Background:** Pneumonectomy is a major surgical option for non-small cell lung cancer (NSCLC). This study evaluates the predictive value of three-dimensional computed tomography (3D-CT)-based lung volume analysis for postoperative function and explores its potential role in preoperative planning, risk assessment, and surgical decision-making. **Methods:** We evaluated 59 NSCLC patients who underwent pneumonectomy. Pre- and 12-month postoperative spirometry results were compared with anatomical and 3D-CT-based predictions. Residual lung expansion was calculated, and patients were grouped by a 3D-CT-derived volume ratio of ≥1.2 or <1.2. **Results:** There was a significant correlation between 3D-CT-based predicted FVC and FEV1 and postoperative spirometric values (*p* < 0.001). The mean residual lung volume expansion ratio was 1.23. Patients with a ratio ≥1.2 had significantly higher postoperative FVC (*p* = 0.028). Lung expansion was observed in 81.4% of cases. Predicted postoperative FVC (*p* = 0.023) and FEV1 (*p* = 0.013) were significantly higher in patients with left pneumonectomy compared to right. **Conclusions:** 3D-CT-based lung volume calculation reliably predicts postoperative function and matches conventional methods. Contralateral lung expansion positively affects respiratory outcomes. Additionally, 3D-CT analysis supports preoperative planning and risk assessment, contributing to more accurate diagnosis and surgical decisions in NSCLC management.

## 1. Introduction

Currently, pulmonary resection is a widely used therapeutic option for malignant and benign lung diseases. Non-small cell lung cancer (NSCLC) is the most common malignancy requiring pneumonectomy [1]. With advancing technology and refined surgical techniques, the 5-year survival rate for patients with early-stage NSCLC after resection has increased to 83% [2,3]. Despite this improvement in survival, a decline in postoperative lung function is inevitable. Approximately 6–12 months after pulmonary resection, the resulting void is typically compensated by mediastinal shift, diaphragmatic elevation, and compensatory expansion of the remaining lung tissue [4]. The extent of functional changes depends on the surgical scope, with reductions in FEV1 reaching up to 9% after lobectomy and 35% after pneumonectomy [5]. In some cases, actual postoperative lung function exceeds the preoperative predicted residual function. This observation is attributed to postoperative compensatory expansion of the residual lung [6,7,8]. Functional and histological analyses indicate that compensatory changes in the residual lung involve alveolar overdistension and increase in functional lung tissue [6,9,10,11].

Current guidelines recommend calculating predicted postoperative (PPO) lung function for all cases during preoperative physiological assessment as a standard metric for evaluating risks of major lung resection [12,13,14]. Conventional methods for estimating post-resection lung function include perfusion scintigraphy (PS) and anatomic segmental counting (ASC) of resected functional units [12,13]. Recent technological advances enable the use of three-dimensional (3D) lung models generated using multidetector computed tomography and simulation software to delineate the vascular and bronchial anatomy before sublobar resections. Preoperative 3D modeling provides more accurate predictions of postoperative residual lung volumes [15]. Several studies have reported that preoperative three-dimensional computed tomography (3D-CT)-based predictions of postoperative pulmonary function yield results comparable to pulmonary function test values [16,17].

The aim of this study was to analyze the morphological and functional reserves of the contralateral lung after pneumonectomy using respiratory function parameters and 3D-CT-based volume calculation. We hypothesized that 3D-CT-based volumetric analysis could not only predict postoperative pulmonary function but also quantify residual lung expansion after pneumonectomy, thereby serving as a complementary tool to conventional estimation methods in preoperative evaluation.

## 2. Materials and Methods

### 2.1. Patients and Study Protocol

**Figure 1:**
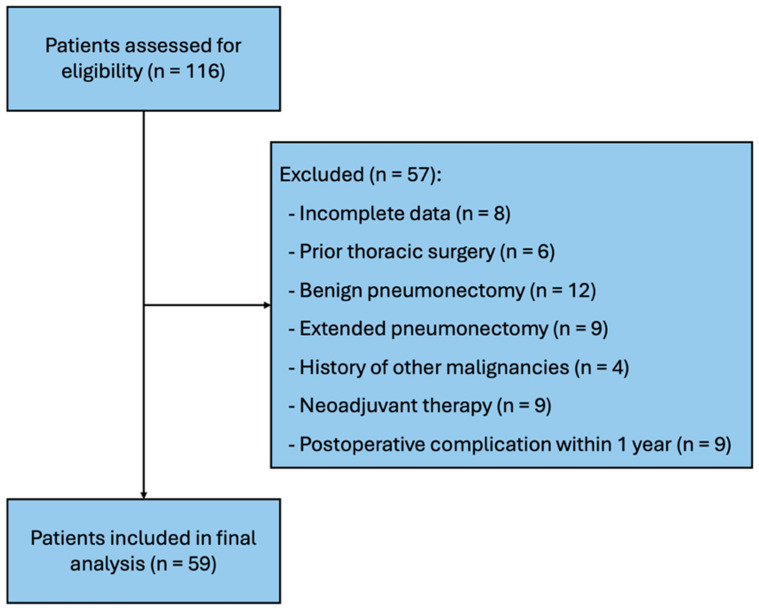
Flowchart of participant inclusion and exclusion.

This retrospective observational study evaluated 59 consecutive Caucasian patients who underwent pneumonectomy for NSCLC at two different centers between January 2019 and 2023. For each patient, the optimal predicted postoperative lung function was determined using anatomic segmental counting and 3D-CT-based lung volume calculation. During this period, patients were excluded if they had incomplete records, history of thoracic surgery, underwent pneumonectomy for benign indications, had an extended pneumonectomy, had history of other malignancies, received neoadjuvant therapy, or developed complications within 1 year. The flow of participant inclusion and exclusion is illustrated in Figure 1. Benign pneumonectomies were specifically excluded due to their low incidence in our dataset and to ensure a homogeneous cohort with comparable oncologic indications and perioperative characteristics. Patient data were analyzed in terms of demographic characteristics, medical history, side of operation, pre- and postoperative high-resolution thoracic 3D-CT–reconstructed lung volumes, pre- and postoperative FVC and FEV1, PPO FVC and FEV1, and postoperative residual lung expansion ratio. A post hoc power analysis demonstrated that the sample size was sufficient to detect statistically significant differences (power > 0.80).

This study was conducted in accordance with the principles of the Declaration of Helsinki. Ethical approval was obtained from the Ethics Committee of Recep Tayyip Erdogan University (Approval No: 2024/156, Date: 28 June 2024).

### 2.2. Computed Tomography Image Acquisition and Postprocessing

CT images were obtained from the hospital database for lung cancer diagnosis, staging, and at the 12th postoperative month. All images were acquired with patients in the supine position during a deep inspiratory breath-hold, using a 16-slice multidetector CT scanner (Toshiba Alexion TSX-034A, Shimoishigomi, Otawara-shi, Tochigi-ken, Japan) with the following parameters: slice thickness, 0.3 mm; reconstruction increment, 0.5 mm; collimation, 16 × 0.75 mm; pitch, 0.8; slice collimation, 0.6 mm; voltage, 120 kVp; reference tube current-time product, 160 mAs; and rotation time, 0.6 s.

**Figure 2:**
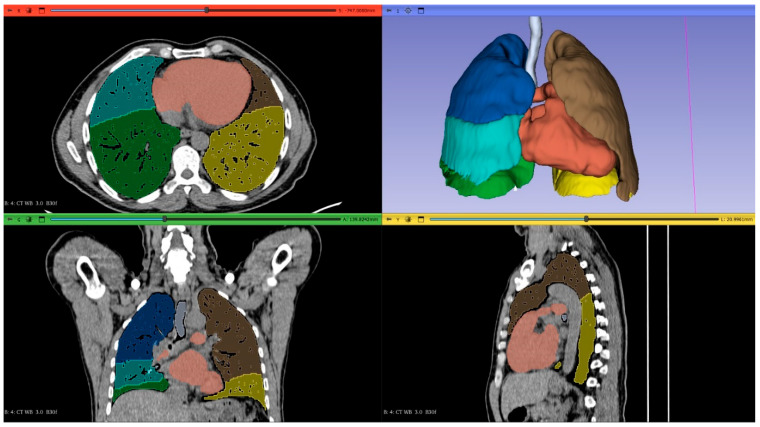
Preoperative 3D−CT volume measurement and visualization of both lungs in a patient scheduled for pneumonectomy.

**Figure 3:**
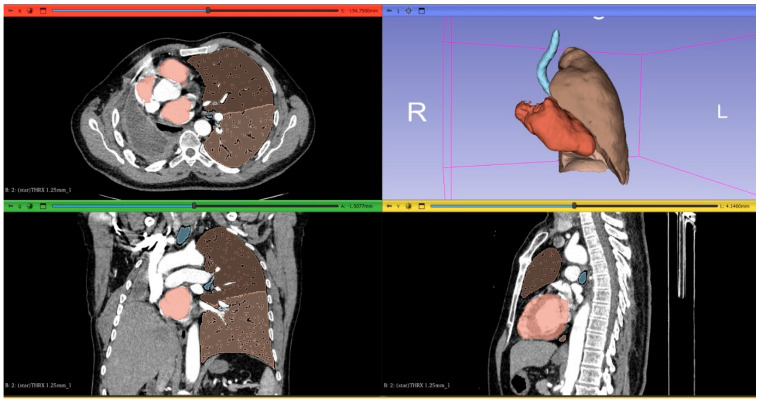
Measurement and visualization of the volume of the residual lung by 3D−CT volume measurement method with the control thorax CT of a patient who underwent pneumonectomy 1 year later.

Thin-section CT DICOM images were transferred to 3D Slicer software (a free, open-source medical imaging platform) for 3D lung volume rendering. A threshold range from −600 to −1024 HU was applied to exclude surrounding soft tissues, large vessels, atelectasis, fibrosis, and intrapulmonary tumors. The volume of segmented lungs at the above threshold was automatically calculated by the software. The volume of both lungs within the −600 to −1024 HU range was defined as the total lung volume. Semi-automated 3D reconstruction of lungs was performed using the software, which also enabled automatic 3D quantification of each lung’s volume and relative volume percentages based on pulmonary landmarks and attenuation thresholds. Figure 2 and Figure 3 show the 3D-CT images of the patients before and after pneumonectomy.

### 2.3. Prediction of Postoperative Lung Function

Pulmonary function tests, including FVC and FEV1, were performed before pneumonectomy and 12 months postoperatively. Predicted postoperative FVC and FEV1 were calculated using the segment counting and 3D-CT lung volumetric methods. In the segment counting method, the predicted values (FVC and FEV1) were calculated using the following equation. The number of resected segments (S) was determined as 10 and 9 for right and left pneumonectomy, respectively [13].

Predicted postoperative FVC or FEV1 value = (preoperative FVC or FEV1 value) × ((19−S) ÷ 19).

In the 3D-CT lung volumetric method, which is a modified version of the previously described method, predicted values were calculated using the following equation [18].

Predicted postoperative FVC or FEV1 value = (preoperative FVC or FEV1 value) × ([residual lung]/[whole lungs]).

Calculations were performed for absolute and percent predicted values of PPO-FEV1 and PPO-FVC.

Using the estimated residual volume (mL) and total postoperative lung volume (mL), the postoperative residual lung expansion ratio was calculated as: postoperative residual lung volume/predicted residual lung volume. Cases were categorized into two groups based on the following residual lung expansion ratio: those with a ratio ≥1.2 and those with a ratio <1.2.

### 2.4. Statistical Analysis

The Statistical Package for the Social Sciences (IBM SPSS; SPSS Inc., Chicago, IL, USA) software Version 21 was used for statistical analyses. The hypothesis of normal distribution of continuous variables was tested by the Kolmogorov–Smirnov test. Continuous variables were expressed as mean ± standard deviation and categorical variables as n,%. Student’s *t*-test was used to compare continuous variables between two groups, and the Chi-square test was used for the comparison of categorical variables. A *p* value of <0.05 was considered statistically significant.

## 3. Results

The cohort included 59 patients (56 men, 3 women; median age: 62 ± 7 years; range: 43 to 92 years) undergoing pneumonectomy for NSCLC. Mean body mass index was 30.5 ± 5.9. Primary lung cancer diagnoses comprised adenocarcinoma (52 cases), and squamous cell carcinoma (7 cases). Left and right pneumonectomy were performed in 40 (68%) and 19 patients (32%), respectively. Demographic characteristics, preoperative spirometric parameters (FEV1, FVC, FEV1/FVC), postoperative spirometric parameters (FEV1, FVC, FEV1/FVC), PPO values based on segment counting (FEV1, FVC, FEV1/FVC), and 3D-CT volumetry-based PPO values (FEV1, FVC, FEV1/FVC) were compared between the right and left pneumonectomy groups (Table 1).

### Postoperative Residual Lung Expansion and Patient Characteristics

**Figure 4:**
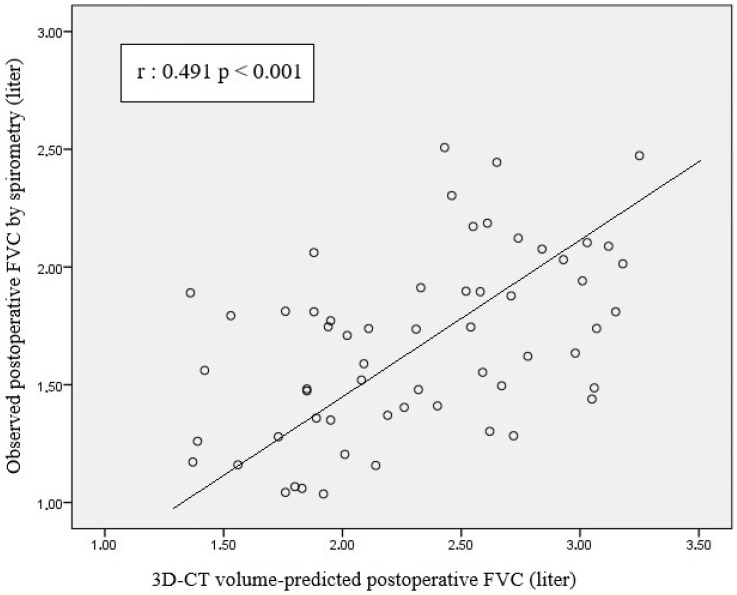
Scatterplot showing the relationship between estimated FVC values calculated based on 3D-CT lung volume and FVC values measured by postoperative spirometry.

**Figure 5:**
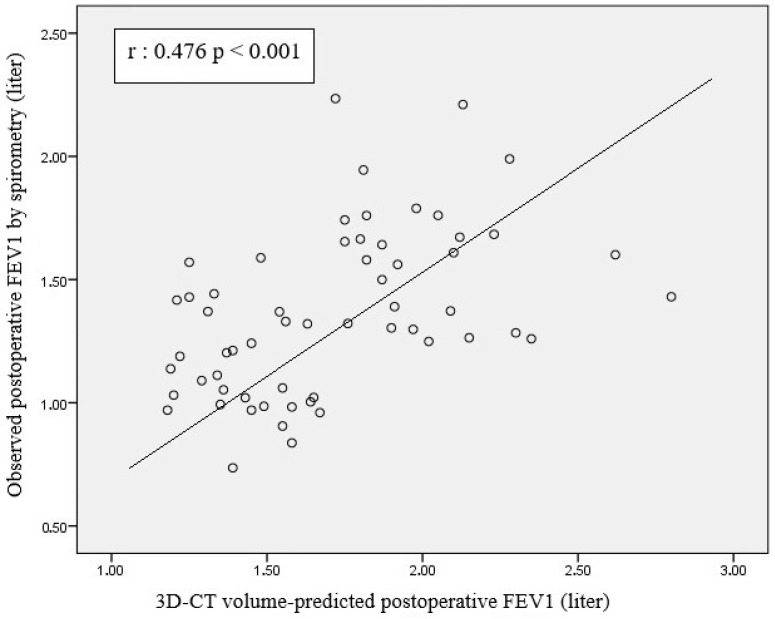
Scatterplot showing the relationship between estimated FEV1 values calculated according to 3D-CT lung volume and FEV1 values measured with postoperative spirometer.

Scatterplot analysis revealed a moderate positive correlation between 3D-CT volumetry-based PPO FVC and postoperative spirometric FVC (r = 0.491, *p* < 0.001) (Figure 4). A significant positive correlation was observed between 3D-CT volumetry-based PPO FEV1 and postoperative spirometric FEV1 (r = 0.476; *p* < 0.001) (Figure 5).

**Figure 6:**
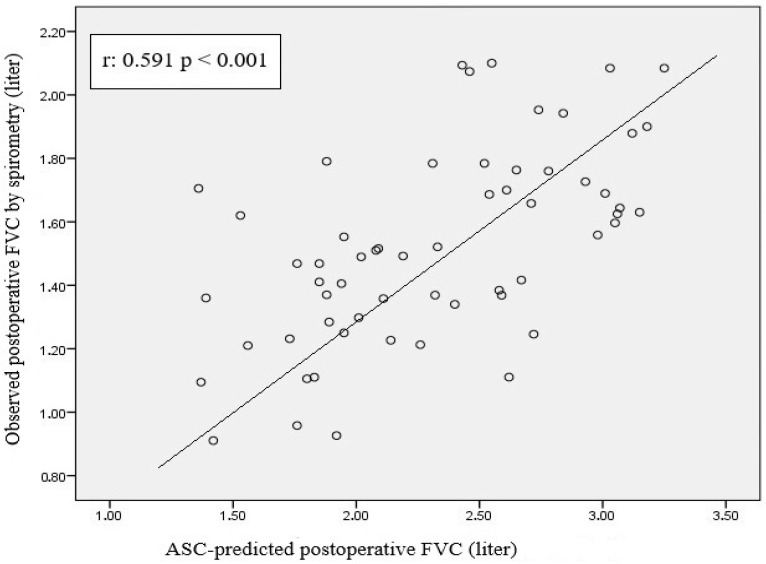
Scatterplot graph showing the relationship between estimated FVC values calculated according to the number of segments and FVC values measured by postoperative spirometer.

**Figure 7:**
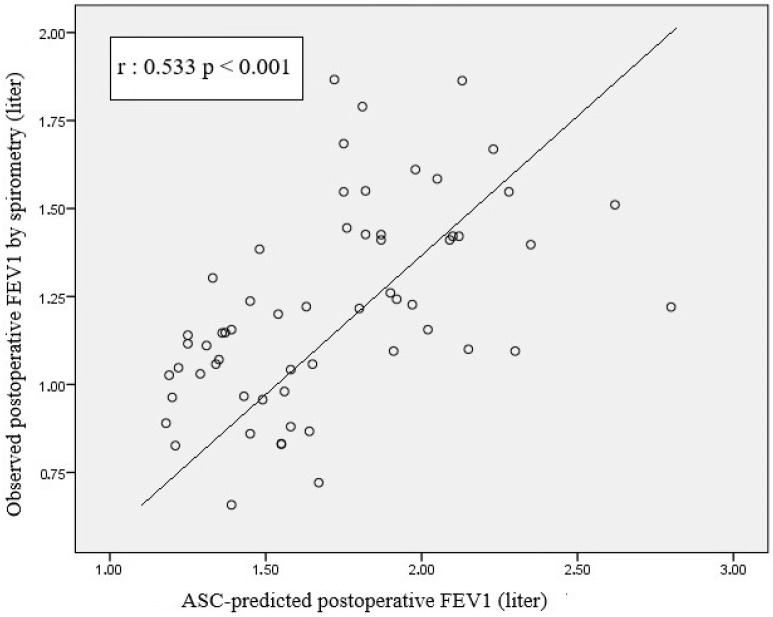
Scatterplot graph showing the relationship between estimated FEV1 values calculated according to the number of segments and FEV1 values measured by postoperative spirometer.

Pearson correlation analysis of the scatterplot between segment count-based PPO FVC and measured postoperative spirometric FVC revealed a significant positive correlation (r = 0.591, *p* < 0.001) (Figure 6). A moderate-to-strong positive correlation was observed between segment count-based PPO FEV1 and postoperative spirometric FEV1 (r = 0.533; *p* < 0.001) (Figure 7).

The mean postoperative residual lung volume expansion ratio was 1.23 (range: 0.69–1.81). The mean residual left lung volume expansion ratio in patients who underwent right pneumonectomy was 1.25. The mean residual right lung volume expansion ratio in patients who underwent left pneumonectomy was 1.21. Thirty-one patients had a postoperative residual lung volume ratio ≥ 1.2, whereas 28 patients had a postoperative residual lung volume ratio < 1.2.

The cases were divided into two groups as left pneumonectomy (n = 40) and right pneumonectomy (n = 19). The left pneumonectomy group demonstrated a significantly higher mean predicted postoperative FVC (*p* = 0.023) and FEV1 (*p* = 0.013) based on 3D-CT volumetry compared with the right pneumonectomy group (Table 1).

The results showed that the group with a postoperative residual lung volume ratio ≥ 1.2 had significantly higher mean postoperative spirometric FVC than the group with a postoperative residual lung volume ratio < 1.2 (*p* = 0.028) (Table 2).

Postoperatively, 48 cases (81.4%) showed an increase in residual lung volume, whereas 11 cases (18.6%) showed a decrease.

According to spirometric measurements performed at 12 months postoperatively, mean FVC loss was 21.8% and mean FEV1 loss was 27.5%.

## 4. Discussion

The findings of this study emphasize the following four points:

(a) There was a significant positive correlation between 3D-CT volumetry-based PPO FVC and FEV1 and postoperative FVC and FEV1 values.
(b) There was a moderate-to-strong positive correlation between segment count-based predicted FVC and FEV1 and postoperative FVC and FEV1 values.
(c) The left pneumonectomy group had higher mean FVC and FEV1 values estimated using postoperative 3D-CT volumetry than the right pneumonectomy group.
(d) Cases with a postoperative residual lung volume ratio ≥ 1.2 had significantly higher mean postoperative FVC value than cases with a ratio < 1.2.

Three-dimensional reconstruction CT has gained popularity among thoracic surgeons for preoperative assessment owing to its intuitive visualization of segmental anatomy [19]. Studies have also shown that volumetric CT provides superior prediction of PPO lung function compared with conventional methods like ASC or PS [12]. The present study compared two methods for predicting postoperative FVC: 3D-CT lung volumetry calculation and the conventional segment-count approach.

Our analysis revealed a moderate positive correlation between 3D-CT-based PPO FVC and postoperative spirometric FVC (r = 0.491, *p* < 0.001). As PPO FVC increased, postoperative spirometric FVC also tended to increase. However, the wide distribution of data points suggests that the accuracy of the PPO FVC in forecasting postoperative FVC is limited. This statistically significant correlation (*p* < 0.001) suggests that calculations based on 3D-CT lung volumetry can be used to predict postoperative pulmonary function; however, variability at the individual patient level should be considered. Despite the presence of a linear trend in the graph, there is significant scatter, which indicates substantial variation when applying PPO-FVC values at the individual level. When the relationship between the PPO-FVC values based on segment counting and postoperative spirometric FVC was examined, Pearson correlation analysis revealed a significant and positive association between the two variables (r = 0.591, *p* < 0.001). The data points showed a more pronounced linear trend, albeit with some scatter, indicating that segment count-based prediction is a robust tool for predicting postoperative FVC, although individual differences should be considered. The high level of statistical significance (*p* < 0.001) supports the fact that this association is not coincidental and presents a clinically reliable relationship. These findings suggest that the calculation based on the number of segments is more accurate and reliable in predicting postoperative pulmonary function than that based on 3D-CT lung volumetry. Nevertheless, 3D-CT volumetry demonstrates clinically relevant accuracy approaching that of segment-based methods. We attribute this to the segment-based method’s more precise representation of the lung’s anatomical and functional units; thus, better representing the residual postoperative lung capacity. The observed dispersion across both methods underscores the influence of individual factors (age, comorbidities, preoperative pulmonary function, and surgical technique) on postoperative FVC. Therefore, for either method to be applied effectively in clinical practice, these patient-specific variables must be considered. Although there is a statistically significant and positive relationship between predicted and actual postoperative-FVC values, the moderate strength of this correlation indicates that additional variables should inform individualized patient management. Without requiring extra measurements or invasive tests, a predicted-FVC value can be calculated from existing anatomical and volumetric data, offering time-saving and cost-based advantages. Thus, preoperative 3D-CT volumetry can provide an early insight into patients’ postoperative respiratory reserve, aiding surgical candidacy assessment and patient counseling. Moreover, the combined use of both methods offers complementary perspectives for a more holistic decision-making process. In cases where the different calculation methods yield discordant results, individualized decision making becomes even more critical. For such patients, supplementary tests (e.g., diffusion capacity measurement or a six-minute walk test) are recommended to obtain supportive functional data. Importantly, diffusion capacity measurement and six-minute walk tests should not be limited to discordant cases but rather considered essential components of comprehensive preoperative evaluation for all patients undergoing lung resection.

In the present study, a significant positive correlation was observed between postoperative FEV1 (L) measured using spirometry and predicted FEV1 values based on 3D-CT lung volumetry after resection (r = 0.476; *p* < 0.001) (Figure 5). The moderate strength of this correlation indicates that the volume-based prediction method has a certain degree of predictive power for postoperative respiratory function. The scattering of data points around the linear regression line demonstrates a positive relationship between the two parameters; however, notable deviations in some patients suggest that the volume-based model may not fully capture individual variability.

Similarly, we observed a moderate-to-strong positive correlation between predicted FEV1 values based on segment counting and postoperative spirometric FEV1 values (r = 0.533; *p* < 0.001) (Figure 7). This result indicates that calculations based on ASC offer a statistically robust method for predicting postoperative respiratory function. The closer clustering of data points around the regression line suggests that this method’s predictive power may be somewhat greater than that of the volume-based approach. The correlation coefficient (r = 0.533) > 0.5 supports a clinically meaningful association between predicted and measured values. When the methods used in the present study to predict postoperative FEV1 values were compared, both demonstrated significant positive correlations. The segment-based prediction method yielded a stronger correlation with r = 0.533, whereas the volume-based method showed a weaker relationship with r = 0.476. These findings suggest that segment-based calculations may be more reliable for forecasting postoperative pulmonary function. Fan et al. observed that the lung volume-based calculation method used to predict postoperative lung function in patients undergoing lung resection demonstrated better accuracy and consistency than the traditional segment-counting method, and they reported it to be a valuable tool for predicting postoperative lung function [20]. However, marked inter-individual differences were observed with both methods, and the data points were found to deviate from the regression line. This suggests that predictions based solely on anatomical or volumetric parameters may be insufficient, and that functional distribution (e.g., ventilation–perfusion ratios) as well as patient-specific factors (e.g., presence of chronic obstructive pulmonary disease, lung reserve, surgical complications) may play a decisive role in the outcomes. Some studies have also reported that while FEV1 predictions are useful for preoperative evaluation, their ability to predict complications is limited when used alone, and they should be supported by diffusion capacity, exercise testing, and clinical assessment [21,22] Although segment-based FEV1 calculations offer greater predictive power for the postoperative period, the limitations of both methods should be taken into account, and a multidisciplinary and comprehensive evaluation approach should be adopted in surgical planning.

Our results demonstrate that 3D-CT volumetry provides better predictive accuracy for postoperative lung function than conventional methods like ASC. Regarding measured postoperative function, volumetric CT-derived predictions achieve similar correlation, precision, and concordance with ASC. We performed comprehensive measurements to predict postoperative pulmonary parameters using volumetric CT, accounting for variable predictive capacity. Compared with traditional segment-based estimations, 3D-CT volumetry may provide a more patient-specific and anatomically precise prediction of postoperative function, particularly in patients with distorted or asymmetrical lung anatomy. The generated predicted postoperative data remain valid for patients considered for lung resection, particularly important given pulmonary resection differentially impacts the lungs’ functional parameters. Documented declines from preoperative values are 30% for FVC and 28% for FEV1 after pneumonectomy versus 13% and 8% after lobectomy, respectively [23]. Bolliger et al. reported a 36% FVC reduction postpneumonectomy versus 6% post lobectomy [24]. In the present study, losses of 21% in FVC and 27% in FEV1 were observed, consistent with the literature.

Postoperative increases in functional residual lung parenchyma are more likely to be observed in young patients [9,11,25]. Although human alveolar proliferation continues postnatally, it typically plateaus during school age. Butler et al. reported functional parenchymal augmentation after pneumonectomy in young adults with lung cancer [9]. These findings suggest that residual lung expansion involves hyperinflation of the remaining lung and an increase in functional lung parenchyma.

Ueda et al. conducted a comparative study on postoperative lung volume changes between lobectomy and segmentectomy and found that the remaining lung expanded more prominently in cases of lobectomy [8]. Mizobuchi et al. observed an increase in lung volume on the contralateral side [11]. In cases involving pneumonectomy, the remaining lung is always the contralateral lung, and as demonstrated in this study, the contralateral lung showed a marked increase in volume indicative of compensatory lung growth (CLG) [11]. In another study by Mizobuchi et al., patients were divided into two subgroups based on the number of resected segments (<10 vs. ≥10) [6]. The authors observed that the subgroup undergoing ≥10 segment resections exhibited a higher postoperative increase in lung volume. Suzuki et al. reported that postoperative recovery rates of lung volume and respiratory function were higher in the lobectomy group than the segmental resection group [26]. Abe et al. demonstrated that postoperative lung expansion was more commonly observed in patients who underwent resection of a greater number of segments [4]. Structural mechanical tension is considered a mechanism for greater CLG in patients with larger resection volumes [27]. In a recent study, Shibazaki et al. reported a residual lung expansion ratio of 1.17 [28] Given that their cohort included 142 lobectomy patients, representing a smaller resection volume than pneumonectomy, this ratio is understandably lower. In the present study, 81.4% of pneumonectomy cases exhibited increased contralateral residual lung volume. Moreover, the mean residual lung volume expansion ratio among our patients was 1.23. These findings demonstrate that even after extensive resections, the contralateral residual lung undergoes significant enlargement. Following pneumonectomy, a large void is created within the thoracic cavity and pressure on mediastinal structures is relieved. As these structures move freely into the empty space, the volume available to the contralateral residual lung indirectly increases. To occupy this space, the contralateral lung hyperinflates and expands volumetrically. Postoperative expansion of the contralateral lung may enhance the ventilatory capacity of the remaining lung tissue, thereby contributing to overall respiratory performance. Additionally, the relaxation of mediastinal structures may facilitate more efficient function of the bronchial and vascular components. However, it should be noted that excessive hyperinflation may impair ventilation–perfusion balance, underscoring the importance of controlled and balanced expansion. While the 12-month follow-up provides a standardized timepoint for evaluation, it should be noted that residual lung expansion, mediastinal shift, and associated physiological adaptations may continue beyond this period. Thus, the presented results reflect an intermediate stage in the dynamic post-pneumonectomy process.

In the present study, the left pneumonectomy group had significantly higher mean PPO FVC and FEV1 values based on 3D-CT volumetry than the right pneumonectomy group (*p* = 0.023 and *p* = 0.013, respectively). This may reflect the larger volumetric capacity of the residual right lung after left pneumonectomy, which could favorably influence outcomes in this group. In addition to anatomical size, this asymmetry is further supported by physiological differences in pulmonary perfusion: under normal conditions, the right lung receives approximately 50–55% of total pulmonary blood flow, while the left receives about 45–50%, due to its smaller vascular network [29]. This perfusion dominance may enhance the compensatory potential of the residual right lung. Conversely, after right pneumonectomy, the heart and mediastinal structures may physically limit expansion of the remaining left lung. Therefore, patients undergoing left pneumonectomy may experience superior postoperative respiratory function and an advantage in functional recovery.

Few studies have directly examined the relationship between postoperative residual lung expansion and pulmonary function [30,31]. Shibazaki et al. found that postoperative residual lung expansion affected postoperative FEV1 [28]. In their lobar analyses, right lobectomies had a pronounced impact on FEV1, with FEV1 after right lower lobectomy being better preserved than after right upper lobectomy. We identified no prior studies focusing specifically on pneumonectomy cases as in our series. Given that pneumonectomy is a major lung resection procedure, we hypothesized that evaluating the effect of residual lung expansion on postoperative respiratory function would be particularly meaningful. In the present study, patients with a postoperative residual lung expansion ratio ≥ 1.2 had a significantly higher mean postoperative spirometric FVC value than those with a ratio < 1.2 (*p* = 0.028). Although mean postoperative FEV1 was also higher in the ≥1.2 group, this difference did not reach statistical significance. These data suggest that greater postoperative residual lung volume expansion may be associated with improved postoperative pulmonary function. This finding implies that volumetric expansion alone may not translate directly into proportional improvements in airflow-related parameters such as FEV1, highlighting the importance of integrating both volume- and flow-based assessments when evaluating postoperative functional recovery.

## 5. Limitations

This study has several limitations. First, it was a single-center, retrospective analysis with a small patient cohort, which limits the generalizability of the findings. A more comprehensive multicenter study is needed to validate these findings. Second, factors that might impede compensatory expansion of the contralateral residual lung, such as complete pleural adhesions or restricted mediastinal mobility, were not evaluated in this study. In addition, postoperative CT scans were not systematically evaluated for parenchymal changes such as fibrosis or emphysematous alterations, which may also influence postoperative lung function. Third, due to the retrospective nature of the study, key physiological parameters such as diffusion capacity measurement and six-minute walk test results were not consistently available and therefore not analyzed. The absence of these functional assessments may limit the physiological and clinical interpretability of the results, particularly in the context of comprehensive preoperative evaluation. Fourth, patients who died or were unable to perform pulmonary function tests due to poor clinical status were excluded, introducing potential selection bias. Finally, the predominance of adenocarcinoma in our study population may limit the generalizability of the findings to other histological subtypes, such as squamous cell carcinoma or neuroendocrine tumors. This reflects the composition of our dataset, in which adenocarcinoma was the dominant diagnosis. Future studies including more histologically balanced or homogeneous groups are needed to determine whether 3D-CT volumetry provides equally reliable predictive values across different tumor types.

## 6. Conclusions

The results obtained in the present study demonstrate that 3D-CT-based lung volume calculation provide predictions of PPO lung function that are as accurate as conventional methods like ASC. With respect to measured postoperative pulmonary function, volumetric CT-based estimates achieve similar correlation, precision, and agreement to those obtained through segment counting. Given that CT is now routinely performed for staging in all patients with suspected lung cancer, 3D-CT-based volumetric calculation can concurrently supply the necessary information to assess operability. The results also demonstrated that the contralateral residual lung volume increases after pneumonectomy and that this residual lung expansion positively influences postoperative respiratory function.

## Figures and Tables

**Table 1:** Patient characteristics of left pneumonectomy and right pneumonectomy groups.

Variable	Left Pneumonectomy (n = 40)	Right Pneumonectomy (n = 19)	*p* Value
Age	63.4 ± 7.7	59.9 ± 10.2	0.151
Preoperative lung volume (mL)	2342 ± 468	2019 ± 348	
Postoperative lung volume (mL)	2771 ± 524	2522 ± 605	
Postoperative residual lung expansion ratio	1.21 ± 0.29	1.25 ± 0.27	0.638
3D-CT predicted FVC (mL)	1745 ± 372	1506 ± 351	0.023
3D-CT predicted FEV1 (mL)	1434 ± 318	1208 ± 317	0.013
Volume-predicted FEV1/FVC (%)	82 ± 9	80 ± 7	0.385
ASC-predicted FVC (mL)	1550 ± 326	1463 ± 248	0.311
ASC-predicted FEV1 (mL)	1250 ± 292	1151 ± 260	0.215
Segment-based FEV1/FVC (%)	81 ± 9	78 ± 7	0.271
Preoperative spirometric FVC (mL)	2849 ± 686	2927 ± 759	0.693
Preoperative spirometric FEV1 (mL)	2279 ± 617	2331 ± 680	0.774
Preoperative FEV1/FVC (%)	80 ± 12	79 ± 7	0.762
Postoperative spirometric FVC (mL)	2328 ± 531	2293 ± 537	0.816
Postoperative spirometric FEV1 (mL)	1743 ± 409	1633 ± 284	0.295
Postoperative FEV1/FVC (%)	76 ± 10	74 ± 14	0.574

*p* < 0.05 is significant.

**Table 2:** Patient characteristics according to postoperative residual lung expansion ratio.

Variable	Postoperative Residual Lung Expansion Rate < 1.2 (n = 31)	Postoperative Residual Lung Expansion Rate ≥ 1.2 (n = 28)	*p* Value
Age (years)	62.7 ± 7.9	61.8 ± 9.4	0.683
Preoperative spirometric FVC (mL)	2739 ± 729	3023 ± 659	0.124
Preoperative spirometric FEV1 (mL)	2154 ± 612	2463 ± 624	0.053
Preoperative FEV1/FVC (%)	79 ± 11	81 ± 10	0.383
ASC-predicted FVC (mL)	1459 ± 310	1591 ± 286	0.096
ASC-predicted FEV1 (mL)	1161 ± 272	1282 ± 288	0.103
3D-CT predicted FVC (mL)	1651 ± 414	1687 ± 343	0.722
3D-CT predicted FEV1 (mL)	1321 ± 347	1405 ± 315	0.340
Volume-predicted FEV1/FVC (%)	80 ± 8	83 ± 8	0.225
Postoperative spirometric FVC (mL)	2174 ± 511	2475 ± 510	0.028
Postoperative spirometric FEV1 (mL)	1634 ± 346	1789 ± 395	0.116
Postoperative FEV1/FVC (%)	77 ± 12	73 ± 11	0.268

*p* < 0.05 is significant.

## Data Availability

All data generated or analyzed during this study are included in this article. The data will be available upon reasonable request (contact persons: omer.topaloglu@erdogan.edu.tr).
